# Integrative analysis of functional genomic annotations and sequencing data to identify rare causal variants via hierarchical modeling

**DOI:** 10.3389/fgene.2015.00176

**Published:** 2015-05-08

**Authors:** Marinela Capanu, Iuliana Ionita-Laza

**Affiliations:** ^1^Memorial Sloan-Kettering Cancer CenterNew York, NY, USA; ^2^Department of Biostatistics, Columbia UniversityNew York, NY, USA

**Keywords:** causal variants, hierarchical modeling, sequencing studies, functional score, CADD score

## Abstract

Identifying the small number of rare causal variants contributing to disease has been a major focus of investigation in recent years, but represents a formidable statistical challenge due to the rare frequencies with which these variants are observed. In this commentary we draw attention to a formal statistical framework, namely hierarchical modeling, to combine functional genomic annotations with sequencing data with the objective of enhancing our ability to identify rare causal variants. Using simulations we show that in all configurations studied, the hierarchical modeling approach has superior discriminatory ability compared to a recently proposed aggregate measure of deleteriousness, the Combined Annotation-Dependent Depletion (CADD) score, supporting our premise that aggregate functional genomic measures can more accurately identify causal variants when used in conjunction with sequencing data through a hierarchical modeling approach.

Identifying a small number of variants most likely to be causal in a specific genetic region (such as a gene) is a challenging problem, but of fundamental importance to the understanding of precise biological mechanisms that influence disease (MacArthur et al., 2014). Rare causal variants, by definition, are only seen a small number of times in any given dataset and therefore are hard to distinguish from the abundant natural variation that occurs in any particular gene. This is a difficult setting, where the causal variants are sparse and tend to be weak as well. Experimental functional studies are the gold-standard, but they are expensive and difficult to implement for large number of variants. Statistical methods to help us distinguish between a true causal variant and a null variant are therefore of great interest.

For those causal variants that are rare but appear at sufficient frequency in a dataset, it is possible to prioritize them over other variants in a gene using genetic (i.e., frequency) data alone. However, for those causal variants that are observed only a few times (e.g., singletons, doubletons etc.), clearly these frequencies on their own are insufficient to provide meaningful risk predictions and no method will perform better than random guessing based on genetic data alone. It is for these variants that functional genomic annotations are essential. Large-scale studies such as the Encyclopedia of DNA Elements (ENCODE) project (ENCODE Project Consortium et al., 2012) provide rich functional genomics annotations that can complement genetic data and help identify likely causal variants. Aggregate measures (Frousios et al., 2013; Johansen et al., 2013; Kircher et al., 2014; Ritchie et al., 2014) that combine information across many annotations in a single measure of deleteriousness for a variant have been shown to outperform individual annotations. The usefulness of such functional measures depends on how well they correlate with pathogenicity. Clearly this correlation will depend on the disease or trait under study and possibly the gene or region of interest (e.g., the degree to which a gene is intolerant to functional variation Petrovski et al., 2013).

It seems natural to expect that combining these two pieces of evidence: (1) the genetic score measuring the statistical evidence for a variant being associated with a disease of interest, and (2) various functional scores for the variant, can enhance the ability to identify rare causal variants. In Supplementary Figure 1 we show three possible outcomes for correctly classifying a true causal variant as likely causal as a function of both genetic and functional scores. Evidently, causal variants with low functional scores and low support from genetic data (e.g., singletons or doubletons) will be impossible to classify correctly. At the other extreme, the causal variants with strong support from both genetic and functional scores will be easy to identify. For causal variants falling in-between these two extremes for which neither the genetic data nor the functional scores on their own provide sufficient evidence of their deleteriousness we believe that integrative methods that combine genetic data with functional scores would be most useful for correctly identifying these variants. Indeed, a recent report by a working group of researchers convened by the NHGRI discussed the challenges in reliably implicating sequence variants in human disease, and highlighted the importance of performing “a combined assessment of the genetic, experimental and informatic support for individual candidate variants” (MacArthur et al., 2014).

Hierarchical models (Gelman et al., 2003) provide a natural approach for such an integrative analysis. They offer a formal statistical framework that can be used in this setting to incorporate sequencing data and functional annotations with the goal of identifying rare causal variants. The strength of the hierarchical modeling approach comes from the fact that it draws upon the information contained in functional annotations to augment the weak information from the case/control frequencies for rare variants, thus improving the risk estimation for individual variants. In our setting, the hierarchical model has two levels: the first level contains the parameters of fundamental interest, namely the relative risks conferred by the individual genetic variants, and also individual-level confounding factors. In the second level, the individual log relative risks of the variants are modeled as linear functions of relevant functional scores. More details on the hierarchical modeling approach and its properties as well as applications to two large population-based case-control studies of melanoma and breast cancer can be found in Capanu et al. (2008), Capanu and Begg (2011), Capanu et al. (2011). A recent application of hierarchical modeling to *VPS13B*, a gene involved in Cohen syndrome and autism can be found in Ionita-Laza et al. (2014).

We conjecture that a hierarchical modeling approach incorporating the sequencing data with an aggregate functional measure more accurately classifies rare causal variants compared to a ranking based solely on the variants' functional score. We focus here on the recently proposed deleteriousness measure, the Combined Annotation-Dependent Depletion (CADD) score or C-score (Kircher et al., 2014), but our method can include any such genomic annotations. We investigate the discriminatory ability of this hierarchical modeling approach and that of the ranking based on the C-scores alone using simulations based on exome sequencing data for 861 individuals (part of the ARRA Autism Sequencing Collaborative project) for one gene, *VPS13B*, containing 166 observed single nucleotide variants (SNVs). We assumed 10% of SNVs to be truly causal variants, with relative risks of 1.1, 2, or 4 for the association of the C-score and the causal status of a variant. We then generated the case-control status following the same data generating mechanism as in Ladouceur et al. (2012). Specifically, for carriers of causal variants we generated a continuous phenotype from a normal distribution with mean μ = 0.5 and a standard deviation of 0.2 while for non-carriers we used a standard normal distribution. This corresponds to a moderate standardized effect size of 0.5 (Cohen, 1988). Note that smaller standardized effect sizes were also examined and the results were qualitatively the same (data not shown). Furthermore, individuals with phenotype values above the median were classified as cases, and the remaining individuals as controls.

The ROC curves for the two different methods are displayed in Figure 1 under different degrees of associations between the causal status and the C-scores and assuming μ = 0.5. Solid curves correspond to ROC curves based on the *z*-values estimated from a hierarchical model in which the C-scores were the solely functional predictor, while dashed curves correspond to rankings based on the C-scores of variants that occurred in at least one of the cases. In all configurations, the hierarchical modeling approach has superior discriminatory ability to the C-score method, with biggest improvements observed when the functional score is weakly associated with the causal status (*RR* = 1.1, blue curves). As the association of the C-scores with the causal status gets stronger, the discriminatory ability of the two methods also improves. Similar trends are observed for larger values of μ such as 1, 1.5, and 2 (data not shown).

**Figure 1:**
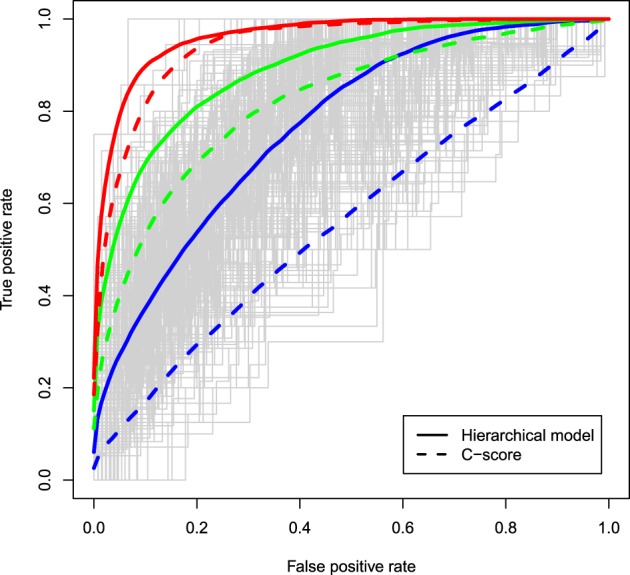
**ROC curves of the *z*-values estimated from a hierarchical model with the C-scores included as a functional predictor (solid curves) and ROC curves based on the ranking of the C-scores of variants carried in at least one of the cases (dashed curves); 10% of variants are assumed truly causal variants; associations between the C-score and the causal status of a variant vary from relative risks of 1.1 (blue), to 2 (green), and 4 (red); μ, the effect size as a function of standard deviations, is assumed to be 0.5; estimates are averaged across 400 simulations**.

In conclusion, aggregate functional measures such as the C-score can identify causal variants more accurately when used in conjunction with sequencing data through a hierarchical modeling approach than when used on their own. Therefore, hierarchical models and other integrative methods are promising tools in this context, and further work in developing such methods is warranted to take full advantage of rich functional annotation data and large scale sequencing studies for many complex diseases in order to understand the nature of the causal variants involved in these diseases.

## Author contributions

MC and II-L wrote and reviewed the manuscript.

### Conflict of interest statement

The authors declare that the research was conducted in the absence of any commercial or financial relationships that could be construed as a potential conflict of interest.
