# Supplementary material for: Integrative analysis of functional genomic annotations and sequencing data to identify rare causal variants via hierarchical modeling
Source: Front Genet. 2015 May 8;6:17. doi: 10.3389/fgene.2015.00176 (PMC4424902; doi:10.3389/fgene.2015.00176)
Supplement: Supplementary file 1 [file Presentation1.PDF]

Supplementary Material for Integrative analysis of functional genomic annotations and sequencing data to identify rare causal variants

Marinela Capanu[1,3] and Iuliana Ionita-Laza[2,3,*]

[1] Memorial Sloan-Kettering Cancer Center, New York, NY 10021

[2] Department of Biostatistics, Columbia University, New York, NY 10032

[3] Equal contribution

[*] Corresponding author: ii2135@columbia.edu

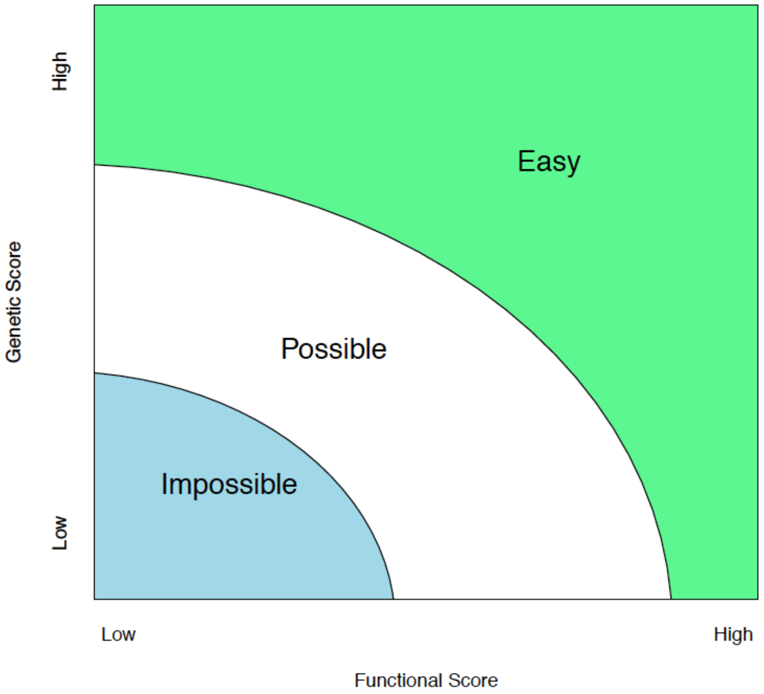

Supplementary Figure 1: The ability to classify a true causal variant as such, as a function of the genetic and functional scores for a variant.
